# Global Metabolic Profiling of *Arabidopsis Polyamine Oxidase 4* (*AtPAO4*) Loss-of-Function Mutants Exhibiting Delayed Dark-Induced Senescence

**DOI:** 10.3389/fpls.2016.00173

**Published:** 2016-02-18

**Authors:** Miren I. Sequera-Mutiozabal, Alexander Erban, Joachim Kopka, Kostadin E. Atanasov, Jaume Bastida, Vasileios Fotopoulos, Rubén Alcázar, Antonio F. Tiburcio

**Affiliations:** ^1^Department of Natural Products, Plant Biology and Soil Science, Laboratory of Plant Physiology, Faculty of Pharmacy, University of BarcelonaBarcelona, Spain; ^2^Max-Planck-Institut für Molekulare PflanzenphysiologiePotsdam-Golm, Germany; ^3^Department of Agricultural Sciences, Biotechnology and Food Science, Cyprus University of TechnologyLimassol, Cyprus

**Keywords:** *Arabidopsis*, polyamines, senescence, spermine, oxidative stress, polyamine oxidases

## Abstract

Early and more recent studies have suggested that some polyamines (PAs), and particularly spermine (Spm), exhibit anti-senescence properties in plants. In this work, we have investigated the role of *Arabidopsis Polyamine Oxidase 4* (*PAO4*), encoding a PA back-conversion oxidase, during dark-induced senescence. Two independent *PAO4* (*pao4-1* and *pao4-2*) loss-of-function mutants have been found that accumulate 10-fold higher Spm, and this associated with delayed entry into senescence under dark conditions. Mechanisms underlying *pao4* delayed senescence have been studied using global metabolic profiling by GC-TOF/MS. *pao4* mutants exhibit constitutively higher levels of important metabolites involved in redox regulation, central metabolism and signaling that support a priming status against oxidative stress. During senescence, interactions between PAs and oxidative, sugar and nitrogen metabolism have been detected that additively contribute to delayed entry into senescence. Our results indicate the occurrence of metabolic interactions between PAs, particularly Spm, with cell oxidative balance and transport/biosynthesis of amino acids as a strategy to cope with oxidative damage produced during senescence.

## Introduction

Polyamines (PAs) putrescine (Put), spermidine (Spd), and spermine (Spm) are nitrogen-containing compounds of low molecular weight known to participate in stress responses (Alcázar et al., 2010; Takahashi and Kakehi, 2010; Minocha et al., 2014; Tiburcio et al., 2014). The polycationic nature of PAs enables their participation in the modulation of cell ion balance as well as in the interaction with negatively charged molecules such as membrane lipids, proteins, and nucleic acids (Schuber, 1989; Cai et al., 2014). Protection of plant cell membranes by PAs has been documented and this might underlie some of the anti-senescence properties reported (He et al., 2002; Liu et al., 2007; Del Duca et al., 2014). However, PAs cannot only be considered as mere polycations stabilizing macromolecules. Evidence indicate that PAs have intrinsic properties and some act as signaling molecules (Minocha et al., 2014; Moschou and Roubelakis-Angelakis, 2014; Tiburcio et al., 2014). Some of the reported anti-senescent effects of PAs have been associated with their ability to act as free radical scavengers and inhibitors of lipid peroxidation (Stoynova et al., 1999; Navakoudis et al., 2007; Yaakoubi et al., 2014). Therefore, the mechanisms of action of PAs seem multiple and additive. As such, the use of omic approaches might be useful for unraveling PA mechanistic processes, and to integrate PAs in the context of global metabolic networks.

Polyamine levels mostly depend on the balance between PA biosynthesis and catabolism. PA catabolism is mediated by two types of amine oxidases: copper-containing amine oxidases (CuAO) and FAD-containing PA oxidases (PAO) (Cona et al., 2006; Angelini et al., 2010). Spd, Spm, and thermospermine (tSpm) are preferential substrates of PAO activity (Takahashi et al., 2010; Fincato et al., 2011; Tavladoraki et al., 2012). PAOs are classified depending on whether they terminally oxidize PAs or catalyze their back-conversion (Angelini et al., 2010; Moschou et al., 2012). PAOs catalyzing PA back-conversion oxidize the carbon at the *exo* side of the N^4^ of Spd and Spm, producing Put and Spd, respectively. *Arabidopsis thaliana* (thereafter referred to as *Arabidopsis*), carries five genes coding for PAOs (*AtPAO1–5*; Takahashi et al., 2010; Fincato et al., 2011). Tissue- and organ-specific expression studies of *AtPAO* gene family members have shown some overlapping patterns but also contrasted differences. This, together with their different substrate specificity, suggests a functional evolutionary diversification of the *AtPAO* gene members (Takahashi et al., 2010). The different subcellular localization of AtPAO proteins may further support this view. AtPAO2–4 are peroxisomal proteins, whereas AtPAO1 and AtPAO5 are predicted to be cytosolic (Takahashi et al., 2010; Fincato et al., 2011, 2012; Kim et al., 2014).

Oxidation of PAs by amine oxidases not only contributes to the regulation of PA homeostasis but also generates products linked to different biological functions (Angelini et al., 2010; Tiburcio et al., 2014). PAs are metabolically linked to reactive oxygen species (ROS) through the production of H_2_O_2_ via PA catabolism (Moschou et al., 2008; Takahashi et al., 2010; Fincato et al., 2011; Ono et al., 2012). Indeed, H_2_O_2_ generated by amine oxidase activity has been shown to contribute to stomatal opening (An et al., 2008), trigger programmed cell death (PCD; Tisi et al., 2011) and γ-aminobutyric acid (GABA) accumulation (Bhatnagar et al., 2002; Mohapatra et al., 2010), which is thought to participate in stress signaling (Bouché et al., 2003; Shelp et al., 2012).

Peroxisomes constitute a very important source of ROS and reactive nitrogen species (RNS). Current data suggest a link between PAs and ROS/RNS in stress signaling (Molassiotis and Fotopoulos, 2011; Filippou et al., 2013; Tanou et al., 2014). However, the relationship between PAs, ROS, and RNS, and their integrated effects in plant physiology are not completely established.

PAO4 exhibits high affinity for Spm oxidation, and transforms via back-conversion Spm into Spd, but not Spd into Put (Kamada-Nobusada et al., 2008; Takahashi et al., 2010; Fincato et al., 2011). Previously, *Arabidopsis pao4* loss-of-function mutants were found to display high Spm and low Spd levels in roots (Kamada-Nobusada et al., 2008). From a signaling perspective, Spm can modify the expression of several genes encoding redox components (Kamada-Nobusada et al., 2008; Mitsuya et al., 2009). Blockage of Spm oxidation by exogenous inhibitors suppressed this transcriptional response, thus suggesting that H_2_O_2_ derived from Spm oxidation underlies this response (Mitsuya et al., 2009). Even though a potential signaling role has been recognized for Spm through transcriptional approaches, global metabolite profiling in engineered genotypes in which Spm levels are endogenously affected are, to our knowledge, not yet reported. Such studies might provide clearer associations between genotypes and stress-tolerance phenotypes, as well as a better integration of PAs in the context of global metabolic networks (Bitrián et al., 2012).

In this work, we have studied the involvement of *AtPAO4* in *Arabidopsis* during dark-induced senescence, through the phenotypic analysis of two independent *pao4* loss-of-function mutant alleles (*pao4-1* and *pao4-2*). We demonstrate that *pao4* mutation leads to delayed dark-induced senescence. Global metabolic profiling of *pao4* mutants and wild-type plants was carried out to investigate mechanisms linked to primary metabolism that underlie the anti-senescent properties. We found that *pao4* mutation promotes the accumulation of hub metabolites in central metabolism and phytohormone biosynthesis, which are known to protect plants against abiotic stress. We also found interactions between PAs and oxidative, sugar, lipid, and nitrogen metabolism. Our results indicate that Spm accumulation modifies the metabolic profile of *Arabidopsis* plants, thus delaying dark-induced senescence.

## Materials and Methods

### Plant Material and Growth Conditions

*Arabidopsis thaliana* accession Columbia-0 (Col-0) was used as wild type (WT) in this study. Seeds were stratified for 3 days in the dark at 4°C and sown in pots containing a mixture of soil and vermiculite (1:1 [v/v]), irrigated with water and Hoagland-based mineral solution and grown at 21°C under long-day photoperiod (16 h of white fluorescent light, photon flux of 70–90 mmol m^-2^ s^-1^). Dark-induced senescence was carried out on adult plants. Fully expanded leaves from 4-week-old plants were used for all analyses. Dark-induced senescence was established essentially as described (Fotopoulos and Kanellis, 2013). In brief, leaves were floated on water in 25 mm-diameter Petri dishes and incubated in the dark at ambient temperature for a period of 4 days.

### Isolation of *pao4* Mutants and Gene Expression Analyses

Total RNA was isolated from 4-week-old *Arabidopsis* leaves using TRIzol (Invitrogen). Total RNA was treated with DNase I (RNase-free; Promega USA) and reverse-transcribed using the SUPERSCRIPT First-Strand Synthesis kit (Invitrogen) following manufacturer’s instructions. PCR from equal amounts of cDNA was performed using *AtPAO4*-specific primers and TaKaRa Ex Taq^TM^. Amplification of the *Arabidopsis Actin 2* gene (AT3G18780.2) (forward primer, 5′-TCACCACAACAGCAGAGCGGGA -3′and reverse primer, 5′-GAAGATGCCCAGAAGTCT -3′) was used for normalization. The PCR conditions were as follows: 96°C 5 min, followed by 35 cycles (5 s at 96°C, 10 s at 64°C, and 40 s at 72°C). PCR products were separated on a 1.0% agarose gel. The analysis was repeated three times with identical results.

The AtPAO4 mutants [AtPAO4 SALK_109229 (Kamada-Nobusada et al., 2008), *pao4-2 in this study*; AtPAO4 SALK_133599 (Liu et al., 2014), named *pao4-1*] were obtained from SALK. The position of the T-DNA insertion in SALK_109229 was confirmed by PCR using a combination of *AtPAO4* specific gene primers (forward, 5′- GGTGGTCATGGTCTAATGGTG-3′and reverse, 5′- GAGAGGCACAGTTGCAGTTTC-3′) and T-DNA primer (SALK-LB 5′-TTTGGGTGATGGTTCACGTAGTGGG-3′). For SALK_133599 we used SALK-LB in combination with *AtPAO4* specific primers, forward 5′- TTCCGATAAGCTTCGTCGTTG -3′ and reverse 5′- TGGAGTCATCCCCGCTAGTTC -3′.

### Polyamine Analyses

Polyamines were analyzed by high-performance liquid chromatography (HPLC) separation of dansyl chloride derivatives. The extraction and determination methods have been previously described (Marcé et al., 1995). The analyses were performed in triplicates from three or more independent experiments.

### Pigments Content

Leaf pigments were extracted from 12 mm leaf disks in dimethyl sulfoxide as described by Richardson (Richardson et al., 2002). Chlorophyll concentrations were determined using the equations described by Sims and Gamon (2002).

### Protein Extraction

Total protein was extracted with phenol, as previously described (Wang et al., 2006). Protein concentration was determined by Bradford (Bio-Rad), diluted to a final concentration of 20 μg/μl, and stored at -20°C. 20 μg of total protein extracts were separated by SDS-PAGE in 12.5% acrylamide gels. Bands were resolved using Colloidal Comassie Brilliant Blue G-250 stain.

### Hydrogen Peroxide and Nitric Oxide Quantification

Hydrogen peroxide was quantified using the KI method, as described by Velikova et al. (2000). Nitrite-derived NO content was measured using the Griess reagent in homogenates prepared with Na-acetate buffer (pH 3.6) as described by Zhou et al. (2005). NO content was calculated by comparison to a standard curve of NaNO_2_.

### Lipid Peroxidation

Lipid peroxidation was determined measuring malondialdehyde (MDA) content resulting from the thiobarbituric acid (TBA) reaction using an extinction coefficient of 155 mM^-1^cm^-1^ as described by Hodges et al. (1999).

### Metabolite Profiling

Metabolite profiling by GC-time of flight (TOF)-MS was performed as previously described (Lisec et al., 2006; Erban et al., 2007). 110 mg of frozen ground homogenized material from rosette leaves was extracted in 360 μL of methanol including internal standard ([^13^C_6_] -sorbitol) at 70°C for 15 min and with 200 μL of chloroform at 37°C for 5 min. The polar fraction was prepared by liquid partitioning with 400 μL of water. An aliquot of 80 μL from the upper polar phase was dried in a Speed Vacuum Concentrator for derivatization by methoxyamination in pyridine (40 mg/mL) and subsequent trimethylsilylation in a final volume of 80 μL. Alkanes were added to pyridine for use as retention index standards. Samples were measured using GC-TOF-MS (LECO Instrumente GmbH, Mönchengladbach, Germany). Chromatograms and mass spectra were processed and evaluated using TagFinder software (Luedemann et al., 2008). Metabolite identification was manually supervised using the mass spectral and retention index collection of the Golm Metabolome Database (Kopka et al., 2005; Hummel et al., 2010). Peak heights of the mass fragments were normalized based on sample fresh weight and internal standard [^13^C_6_]-sorbitol.

Metabolic implication of reported altered metabolites in this work, further classification and simplified metabolic maps were made by the use of public database KEGG (Kanehisa and Goto, 1999; Kanehisa et al., 2014) and AraCyc developed by Plant Metabolic Network project (PMN; Mueller et al., 2003; Chae et al., 2012).

### Statistical Analyses

Statistical analyses were performed using IBM^®^ SPSS^®^ Statistics V.22. Biochemical and physiological damage measurements were subjected to ANOVA. Significant differences between individual means were determined using Tukey’s HSD (Honestly significant difference) pairwise comparison test at the 5% confidence level. Data from metabolomics were analyzed and heat maps obtained from MeV: MultiExperiment Viewer v.4.9 (Saeed et al., 2003).

## Results

### Isolation of *pao4* Mutants

**FIGURE 1:**
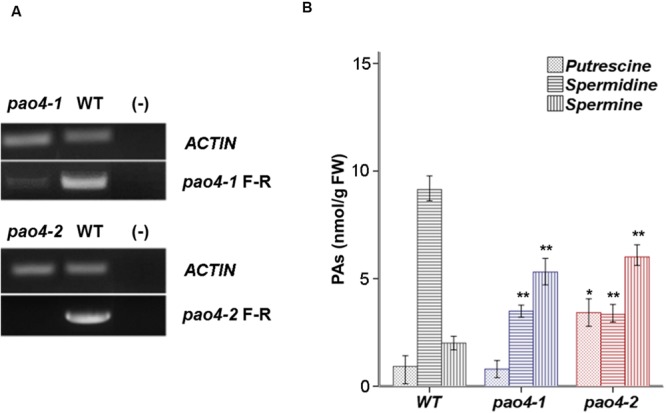
***AtPAO4* expression and PA levels. (A)** *PAO4* gene expression in *pao*4 mutants compared with wild-type. **(B)** Free levels of Put, Spd, and Spm in 4 week-old pao4 mutants grown under optimal growth conditions. Values represent the mean of at least five biological replicates ±SD. Asterisks indicate values which are significally different from the wild-type, as determined by both one-way ANOVA and Tukey HSD *post hoc* tests (*P* < 0.05).

Two independent *AtPAO4* (*At1g65840*) T-DNA insertion mutants (*pao4-1* and *pao4-2*) were isolated that carried single T-DNA insertions. *pao4-2* exhibited no expression of *PAO4*, consistent with a loss-of-function mutation. Conversely, *pao4-1* exhibited residual *AtPAO4* expression and thus resulted in a knock-down mutation (**Figure 1A**). No obvious phenotypical differences were observed between *pao4-1, pao4-2*, and wild-type genotypes under optimal growth conditions, which is in agreement with previous reports (Kamada-Nobusada et al., 2008; Liu et al., 2014). Both *pao4-1* and *pao4-2* mutant alleles were used throughout the experiments.

### PA Levels in *pao4* Mutants

The levels of free Put, Spd, and Spm levels were analyzed in 4 weeks-old *pao4-1, pao4-2* and wild-type plants. PA analyses indicated that both *pao4* mutants accumulated up to 10-fold higher levels of Spm than the wild-type, consistent with Spm being the preferential substrate of PAO4 activity. Conversely, both *pao4-1 and pao4-2* mutants exhibited lower Spd levels than the wild-type (**Figure 1B**). The levels of Put were only increased in *pao4-2* and not in *pao4-1*, probably as result of the residual *PAO4* expression in the latter. We concluded that accumulation of Spm and dampening of Spd levels are common metabolic hallmarks of *pao4-*1 and *pao4-2*.

### Dark-Induced Senescence in *pao4* Mutants

**FIGURE 2:**
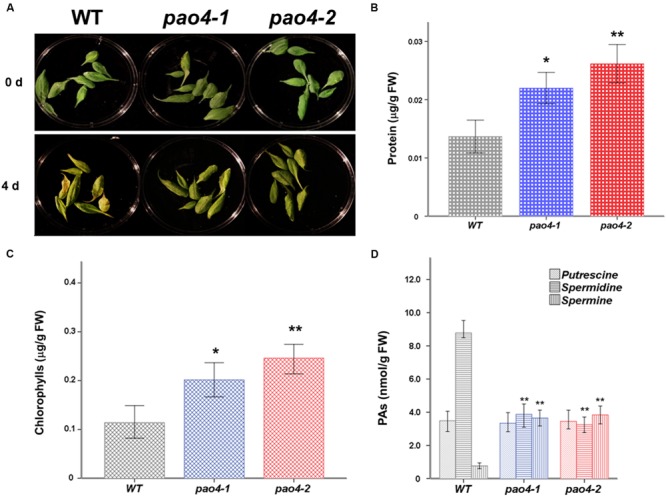
**Effect of dark-induced senescence in wild-type and *pao4* mutants. (A)** Macroscopic observation of wild-type and *pao4* leaves after dark-induced treatment. **(B)** Total protein levels in *pao4* senescent leaves after dark-induced senescence treatment. **(C)** Chlorophyll contents in *pao4* senescent leaves. **(D)** Free PAs (Put, Spd, and Spm) levels in senescent *pao4* leaves. Each value represents the mean of at least five independent biological replicates ±SD. Asterisks indicate values which are significally different from the wild-type, as determined by both one-way ANOVA and Tukey HSD *post hoc* tests (*P* < 0.05).

We investigated the differential response of *pao4* mutants and wild-type plants to early senescence induced by dark treatment. For this, detached mature leaves from 4 week-old *pao4* mutants and wild-type plants grown under optimal conditions were used. No differences in size, senescence status (determined by total chlorophyll and protein levels) or turgor were visible between leaves of the wild-type and *pao4* mutant before the dark-induced treatments (data not shown). Interestingly, both *pao4-1* and *pao4-2* mutants evidenced signs of delayed senescence after 4 days of continuous dark treatment (**Figure 2A**). Total protein levels were measured to quantify the extent of senescence delay induced by *PAO4* mutation. Protein levels were significantly higher in *pao4-1* and *pao4-2* than the wild type, thus suggesting a lower rate of protein degradation consistent with delayed senescence (**Figure 2B**). Quantification of chlorophylls in *pao4-1* and *pao4-2* further supported these observations (**Figure 2C**), suggesting that *pao4* mutation leads to delayed dark-induced senescence.

Polyamine levels were determined during senescence in *pao4* mutants and wild-type. Levels remained constant for most PAs throughout the induced senescence, except for Spd levels, which dropped in *pao4* from fivefold lower than the wild-type under basal conditions to 10-fold lower than the wild-type after senescence treatment (**Figure 2D**).

### H_2_O_2_, MDA, and NO Levels in *pao4* Mutants During Dark-Induced Senescence

**FIGURE 3:**
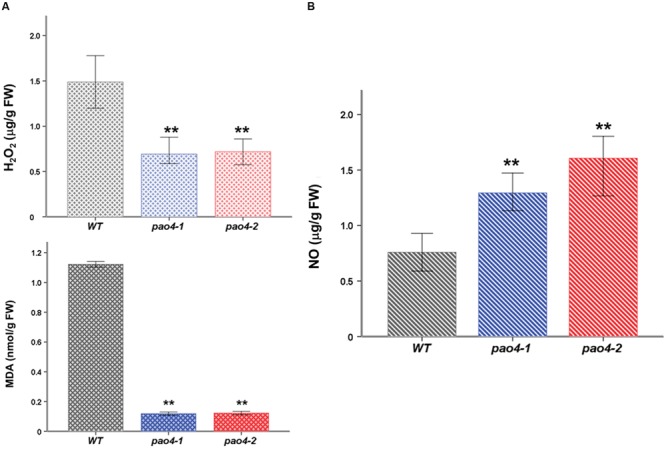
**H_2_O_2_, MDA, and NO levels in *pao4* mutants and wild-type after dark-induced senescence treatment. (A)** H_2_O_2_ and MDA levels **(B)** NO levels. Free radical levels were determined after dark-induced senescence treatment. Values represent the mean of at least five biological replicates ±SD. Asterisks indicate values which are significally different from those of the corresponding wild-type plants, as determined by both one-way ANOVA and Tukey HSD *post hoc* tests (*P* < 0.05).

Reactive oxygen species and RNS are important players of the oxidative and nitrosative response that exhibit contrasted effects on senescence. While ROS generally promote senescence (Khanna-Chopra, 2012), RNS might underlie anti-senescence effects (Niu and Guo, 2012; Liu and Guo, 2013). We measured H_2_O_2_ and NO levels in *pao4-1, pao4-2* and wild-type plants after dark-induced senescence (**Figure 3**). Both *pao4* mutants exhibited lower H_2_O_2_ levels than the wild-type plant after the senescence treatment, thus suggesting the enhancement of the antioxidative machinery in *pao4* (**Figure 3A**). Consistent with these observations, the levels of MDA (a measurement of membrane damage by lipid peroxidation) were significantly lower in *pao4* than the wild-type (**Figure 3A**). Interestingly, the levels of NO exhibited an opposite pattern and accumulated in *pao4* compared with the wild-type (**Figure 3B**). We concluded that ROS production induced by senescence is restricted in *pao4* mutants, whereas NO production is stimulated.

### Metabolomic Profiling of *pao4* Mutants Under Basal Conditions

**FIGURE 4:**
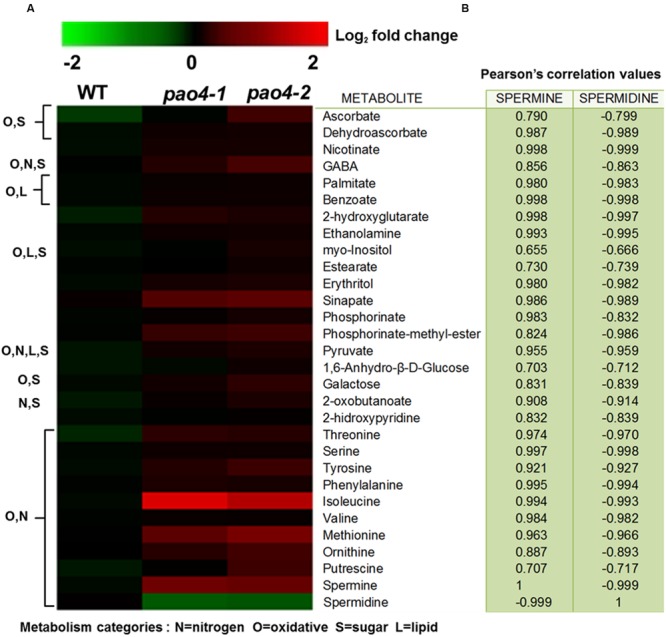
**Heat-map representation of up-regulated metabolites in *pao4* compared with wild-type under control conditions. (A)** Metabolite levels were determined in 4 week-old plants and are expressed as log_2_ relative to wild-type. **(B)** Pearson’s correlation values (r) related to Spm and Spd. Values were obtained from at least four independent biological experiments (*P* < 0.05).

In order to analyze the metabolic consequences of *PAO4* loss-of-function on primary metabolism, we performed GC-TOF/MS metabolomic profiling (Erban et al., 2007; Allwood et al., 2011) in 4-week-old *pao4* mutants and wild-type plants grown under optimal conditions in the absence of stress, referred to as ‘basal’ conditions. Primary metabolite profiling identified a total of 75 metabolites, 37 of which did not show significant differences respect to the wild-type (Supplementary Table S1). From the remaining 38 metabolites, 28 were increased (**Figure 4**) and 10 decreased in *pao4* compared to the wild-type (Supplementary Table S2). Most down-regulated metabolites could not be classified into metabolic groups, because their chemical structure is unknown (Supplementary Table S2). Up-regulated metabolites could be sorted into four major metabolic categories belonging to oxidative and nitrogen metabolism, sugars and lipids. However, many metabolites were shared between categories (**Figure 4A**). Increased metabolites in *pao4* included sugars (galactose), sugar alcohols (*myo*-Inositol, erythritol), ethanolamine and many amino acids (Ser; aromatic amino acids Phe and Tyr; precursors of PAs Orn and Met; branched-chain amino acids Ile and Val). Indeed, amino acids represented the largest group of up-regulated metabolites in *pao4* under basal conditions (**Figure 4A**). Other important upregulated metabolites included pyruvate, which is a crucial hub metabolite, GABA, which is suggested to participate in stress responses, and ascorbate/dehydroascorbate (ASC/DHA), which are important metabolites involved in antioxidant defense pathways. Pearson’s correlation analyses indicated the occurrence of strong positive correlations between Spm and up-regulated metabolites, but negative correlations with Spd (*P* < 0.05; **Figure 4B**). Based on these analyses, we conclude that *pao4* mutants exhibit constitutive accumulation of several amino acids and important stress protection metabolites, and this associates with higher Spm levels and/or Spm/Spd ratios.

### Metabolomic Profiling of *pao4* Mutants After Dark-Induced Senescence

**FIGURE 5:**
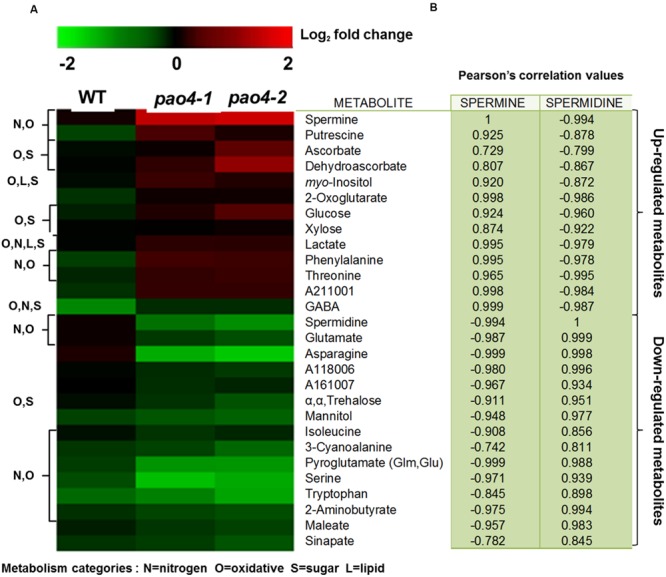
**Heat map of *pao4* mutants altered metabolite pools after dark-induced senescence. (A)** Metabolite levels were determined from detached leaves of 4 week-old plants grown as described (see Materials and Methods). Values represent log_2_-transformed fold-changes relative to the wild-type. The first group (top) represents up-regulated metabolites in pao4 compared with wild-type, and the second group (bottom) down-regulated metabolites in the same comparison. Altered metabolites were detected with MeV tool V.4.9 by rank product statistical test (*P* < 0.05). **(B)** Pearson correlation values (r) related to Spm and Spd, values were obtained from at least four independent biological experiments (*P* < 0.05).

Metabolomic profiling after dark-induced senescence in *pao4* and wild-type leaves identified a total of 103 metabolites (**Figure 5A** and Supplementary Table S3), 28 of which exhibited significant differences between *pao4* and wild-type senescent leaves (**Figure 5A**). Among these, 13 metabolites were up-regulated and 15 down-regulated in *pao4* compared to the wild-type (**Figure 5A**). 8 of the 13 up-regulated metabolites were already increased in *pao4* compared to the wild-type under basal conditions (**Figures 4A** and **5A**). Such constitutively up-regulated metabolites were the PAs Put and Spm, antioxidative metabolites ASC/DHA, *myo*-Inositol, GABA and the amino acids Thr and Phe. Among up-regulated metabolites exclusively induced after senescence treatment in *pao4*, and not in the wild-type, we identified sugars (glucose and xylose) and the TCA cycle intermediate 2-oxoglutarate (**Figure 5A**).

Down-regulated metabolites in senescent *pao4* leaves were amino acids involved in senescence signaling such as Glu, pyroglutamate, Trp, Asn, and 3-Cyanoalanine (**Figure 5A**). The decrease in Glu and Asn is associated with late senescence partly because Asn and 3-Cyanoalanine are products of the cyanide detoxification pathway induced by ethylene biosynthesis (Diaz et al., 2005). Other molecules involved in glucose biosynthesis/degradation, such as α,α, trehalose were down-regulated in *pao4.*

A strong positive correlation was found between up-regulated metabolites in senescent *pao4* leaves and Spm levels, but negative correlations with Spd (*P* < 0.05; **Figure 5B**). Conversely, down-regulated metabolites showed an opposite pattern of strong positive correlation with Spd but negative with Spm, suggesting that homeostasis of these PAs may be relevant in the response to senescence (*P* < 0.05; **Figure 5B**).

## Discussion

The identification of metabolic networks in which PAs are integrated is a necessary step to elucidate potential mechanisms underlying PA-triggered stress protection (Shi and Chan, 2014). Here, we report that loss-of-function mutations in *PAO4*, a member of the five *Arabidopsis AtPAO* gene family, leads to delayed dark-induced senescence and this associates with higher Spm and/or lower Spd/Spm ratios.

Accumulation of Spm in *pao4* mutants (**Figure 1B**) is consistent with the reported higher affinity of PAO4 enzyme toward Spm (Kamada-Nobusada et al., 2008; Takahashi et al., 2010; Fincato et al., 2011). Given the previously reported anti-senescence properties of Spm in plants and animals (Pandey et al., 2000; Serafini-Fracassini et al., 2010; Del Duca et al., 2014; Moschou and Roubelakis-Angelakis, 2014), and the high Spm levels in *pao4-1* and *pao4-2* mutants, current findings suggest that the delayed *pao4* senescence may be associated with the endogenous Spm levels. However, because *pao4* mutants also exhibit lower Spd levels, it cannot be completely ruled out that the Spd/Spm ratio may modulate this response. In any case, global metabolic analyses in both *pao4* mutants indicated that primary metabolism is intricately connected with PA metabolism, and this is differentially regulated in *pao4* under senescence conditions. Our results indicate that loss of PAO4 functionality is beneficial to prevent senescence under dark-inductive conditions.

Global metabolite analyses in *pao4* mutants under basal conditions (**Figure 4**) identified amino acids as the largest group of metabolites which were up-regulated, compared with wild-type plants. Up-regulated amino acids included PA precursors (Met and Orn), branched-chain amino acids, aromatic and polar uncharged, which are essential for post-translational modifications. In addition, most altered amino acids were either involved in day/night cycle transitions (Gibon et al., 2006) or adaptation to extended dark conditions (Gibon et al., 2006, 2009). Spm has previously been shown to reprogram the oxidative status of citrus plants exposed to salt stress, and to increase the ASC redox state (Tanou et al., 2014). In this study, the metabolic profile of *pao4* suggests the constitutive enhancement of anti-oxidative mechanisms, mainly through the accumulation of ASC/DHA, nicotinate and sinapate, which are essential metabolites in the maintenance of anti-oxidative capacity (Hashida et al., 2010; Wang et al., 2010; Foyer and Noctor, 2011; Gallie, 2012).

*pao4-1* and *pao4-2* exhibited accumulation of metabolites in central metabolism and signaling hubs under basal conditions. Such metabolites included pyruvate and *myo*-Inositol, which is involved in sugar and phospholipid signaling (Gillaspy, 2011; Williams et al., 2015). *AtPAO4* loss-of-function also led to the up-regulation of nitrogen-mobilization molecules, such as GABA (Bitrián et al., 2012; Shelp et al., 2012). The role of GABA during stress remains unclear. However, GABA has been proposed to act as a signaling molecule that coordinates the C:N balance in challenging environments, such as prolonged dark conditions (Buchanan-Wollaston et al., 2005). GABA also serves as nitrogen-storage molecule during nitro-oxidative stress (Tanou et al., 2012). Overall, the metabolic profile of *pao4* mutants under basal conditions is consistent with a prime-like status, in which the antioxidant machinery is pre-activated and GABA accumulates. It is therefore suggested that Spm and/or low Spd/Spm ratio triggers pre-acclimation to stress in *Arabidopsis*.

Subsequently, mechanisms underlying the *pao4* anti-senescence phenotype from a metabolic perspective were investigated. The levels of H_2_O_2_ and NO were determined in wild-type and *pao4* mutants after dark treatment. Interestingly, delayed senescence in *pao4* correlated with significant increases in NO levels (**Figure 3B**), which is a pattern consistent with previous observations (Niu and Guo, 2012; Liu and Guo, 2013). Conversely, the levels of H_2_O_2_ were lower in *pao4* than wild-type plants (**Figure 3A**), which is in agreement with promotion of the ASC/DHA cycle in *pao4* (**Figure 4A**) and supports previous findings in which ROS inhibition leads to delayed senescence in tobacco and wheat (Hui et al., 2012; Fotopoulos and Kanellis, 2013; Tian et al., 2013). NO might be an inductive element of the oxidative response after stress imposition (Linka and Theodoulou, 2013; Corpas and Barroso, 2014). It can be hypothesized that priming by Spm confer a more intense dark-induced stress response involving NO signaling.

Compared with the wild-type, most metabolites altered by dark in *pao4* were related to oxidative and nitrogen metabolism (**Figure 5**). Down-regulated metabolites in dark-treated *pao4* were amino acids and compounds involved in their metabolism (**Figure 5A**). This pattern is consistent with high nitrogen mobilization in *pao4* induced by senescence (Soudry et al., 2005). Indeed, interactions have been observed between PA and amino acid metabolism during senescence in *Arabidopsis* (Mattoo et al., 2010; Watanabe et al., 2013). NO is also known to be involved in the regulation of free amino acid levels during the stress response by induction of the γ-glutamyl cycle for GSH biosynthesis (Innocenti et al., 2007), and through modulation of proteolytic mechanisms such as autophagy or the TOR pathway in *Arabidopsis* and other species (López-Berges et al., 2010; Tripathi et al., 2013). Some down-regulated amino acids by dark-induced senescence in *pao4* have important implications in senescence signaling. As such, Glu influences adaptation to dark periods in *Arabidopsis* (Gibon et al., 2009). Glu is also a product of glutathione catabolism along with pyroglutamate (Ohkama-Ohtsu et al., 2007, 2008), which is also involved in mitochondrial reassembly during oxidative stress (Obata et al., 2011) and GABA formation (Soudry et al., 2005; Watanabe et al., 2013). Recent evidence also indicates that increases in nitrogen assimilation favors GSH biosynthesis with concomitant decreases in pyroglutamate and Glu levels (Paulose et al., 2013).

The above data suggest the potential modulation of GSH homeostasis by PAO4 activity, which conditions Spm or Spd/Spm ratio. Metabolite profiling suggests the occurrence of a Spm-triggered oxidative response involved in the maintenance of the redox status throughout modulation of amino acid transport and recycling. Trp is a main precursor of the phytohormone indole-3-acetic acid (IAA; Zhao, 2014), and it participates in plant development and dark-induced senescence signaling (Van der Graaff et al., 2006). Asn and 3-cyanoalanine are products of cyanide detoxification pathway (Piotrowski et al., 2001), which is activated after the final biosynthetic reaction of ethylene (Yamagami et al., 2003). Both Asn and 3-cyanoalanine are considered as senescence markers (Van der Graaff et al., 2006; Watanabe et al., 2013). Cross-talk between PAs and hormones such as ethylene and IAA has been reported, but the molecular nature of such interactions remains elusive (Bitrián et al., 2012). Because *pao4* mutants display lower levels of 3-cyanoalanine, Asn and Trp, it is suggested that high Spm levels might promote delayed entry into dark-induced senescence through inhibition of ethylene biosynthesis, although this requires further investigation.

Aromatic and branched-chain amino acids have been shown to act as alternative electron donors for mitochondrial respiration during the stress response, in a process whereby the hydrolysis of 2-hydroxyglutarate (2-HG) produces 2-oxoglutarate (2-OG) with concomitant release of electrons donated to ubiquinol via the ETFQO complex (Ishizaki et al., 2005; Araújo et al., 2010, 2011; Obata et al., 2011). Interestingly, Phe, 2-HG, and 2-OG were increased in *pao4* mutants compared with wild-type plants, thus suggesting that Spm promotes the alternative electron donor pathway for mitochondrial respiration (**Figure 5**). In support to this view, an Spm-induced signaling pathway leading to mitochondrial dysfunction has previously been reported during biotic stress in tobacco and *Arabidopsis* (Takahashi et al., 2004; Mitsuya et al., 2009). Therefore, it seems reasonable that increases in Spm and NO might enhance mitochondrial energy production after dark-induced senescence.

Other molecules involved in glucose biosynthesis/degradation and enhancement of oxidative burst were also identified, such as α,α, Trehalose (O’Hara et al., 2013). This metabolite has emerged as a redox signaling molecule with a proposed role during stress and senescence (Fernandez et al., 2010; Krasensky et al., 2014). Trehalose degradation confers drought tolerance by producing glucose (Van Houtte et al., 2013), a pattern which has also been observed during dark-induced senescence (Buchanan-Wollaston et al., 2005; Gibon et al., 2006), and is consistent with the increase in glucose levels observed in *pao4* after dark treatment (**Figure 5**).

Furthermore, increases in xylose observed in dark-treated *pao4* plants suggest activation of the phosphate-pentose pathway, which is reported to be up-regulated in *Arabidopsis* roots after oxidative stress imposition (Lehmann et al., 2009) as a source of reducing equivalents in peroxisomes for GSH biosynthesis (Corpas et al., 2009). Increased lactate was also found, which is consistent with a link between sugar and pyruvate-related amino acid metabolism.

**FIGURE 6:**
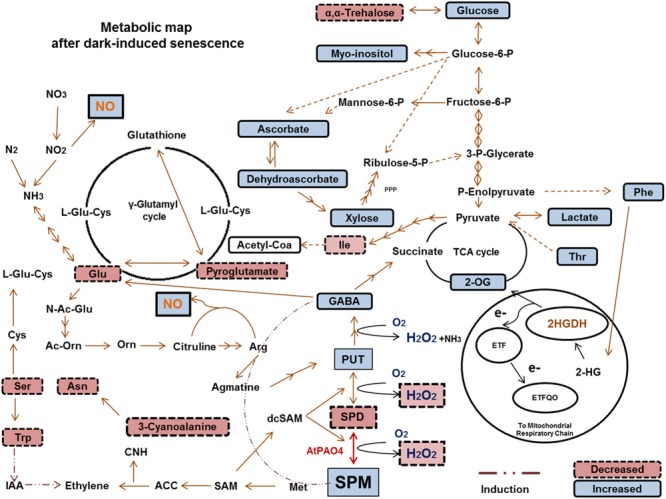
**Metabolic interactions between PAs and primary metabolism, and the observed increases/decreases in the comparisons between pao4 and wild-type under dark-induced senescence.** 2-HG, 2-Hydroxyglutarate; 2HGDH, 2-Hydroxyglutarate Dehydrogenase; ETF, Electron-transfer flavoprotein; ETFQO, Electron-transfer flavoprotein: Ubiquinone oxidoreductase; 2-OG, 2-Oxo-glutarate; SAM, S-Adenosylmethionine; dcSAM, Decarboxilated S-Adenosylmethionine; ACC, Aminocyclopropane Carboxilic Acid; CNH, Hydrogen Cyanide; IAA, Indole-3-Acetic Acid; SA, Salycilic Acid; JA, Jasmonic acid N-Ac-Glu; N-Acetyl-L-Glutamate; Ac-Orn, Acetylornithine; L-Glu-Cys, L-Glutamylcysteine.

Overall, we provide a global view of metabolic changes affected by *PAO4* mutation in *Arabidopsis*, which are associated with delayed entry into dark-induced senescence (**Figure 6**). Current findings suggest that the delayed *pao4* senescence may be associated with high Spm levels, reduced ROS production and increased NO levels. Furthermore, our results point to an important role of Spm as a ‘signaling’ metabolite promoting stress protection through metabolic connections involving ASC/GSH redox state modifications, changes in sugar and nitrogen metabolism, cross-talk with ethylene biosynthesis and mitochondrial electron transport chain modulation, all of which are involved in the nitro-oxidative response after stress imposition.

## Author Contributions

Performed research: MS-M, AE, KA; Analyzed the data; MS-M, AE, JK, VF, RA, AT; Designed research: JK, JB, VF, RA, AT; Wrote the paper: MS-M, VF, RA, AT.

## Conflict of Interest Statement

The authors declare that the research was conducted in the absence of any commercial or financial relationships that could be construed as a potential conflict of interest.

The reviewer PT and handling Editor declared their shared affiliation, and the handling Editor states that the process nevertheless met the standards of a fair and objective review.
